# Associations between frequency of food shopping at different store types and diet and weight outcomes: findings from the NEWPATH study

**DOI:** 10.1017/S1368980016000355

**Published:** 2016-03-09

**Authors:** Leia M Minaker, Dana L Olstad, Mary E Thompson, Kim D Raine, Pat Fisher, Lawrence D Frank

**Affiliations:** 1 Propel Centre for Population Health Impact, University of Waterloo, 200 University Avenue West, Waterloo, Ontario, Canada, N2L 3G1; 2 Centre for Physical Activity and Nutrition Research, School of Exercise and Nutrition Sciences, Faculty of Health, Deakin University, Burwood, Victoria, Australia; 3 Statistics and Actuarial Sciences, Faculty of Mathematics, University of Waterloo, Waterloo, Ontario, Canada; 4 School of Public Health, University of Alberta, Edmonton, Alberta, Canada; 5 Region of Waterloo Public Health Department, Waterloo, Ontario, Canada; 6 School of Community and Regional Planning, University of British Columbia, Vancouver, British Columbia, Canada

**Keywords:** Food shopping, Diet quality, Weight status, Food retail sources

## Abstract

**Objective:**

The present study aimed to: (i) examine associations between food store patronage and diet and weight-related outcomes; and (ii) explore consumer motivations for visiting different types of food store.

**Design:**

A stratified probability sample of residents completed household and individual-level surveys in 2009/2010 on food purchasing patterns and motivations, dietary intake, waist circumference (WC), weight and height. Diet quality was calculated using the Healthy Eating Index for Canada from a subset of participants (*n* 1362). Generalized estimating equations were created in 2015 to examine how frequency of patronizing different types of food store was associated with diet quality, intake of fruits and vegetable, mean intake of energy (kcal) sodium and saturated fat, WC and BMI.

**Setting:**

Three mid-sized urban municipalities in Ontario, Canada.

**Subjects:**

A representative sample of residents (*n* 4574).

**Results:**

Participants who shopped frequently at food co-ops had significantly better diet quality (*β*=5·3; 99 % CI 0·3, 10·2) than those who did not. BMI and WC were significantly lower among those who frequently shopped at specialty shops (BMI, *β*=−2·1; 99 % CI −3·0, −1·1; WC, *β*=−4·8; 99 % CI −7·0, −2·5) and farmers’ markets (BMI, *β*=−1·4; 99 % CI −2·3, −0·5; WC, *β*=−3·8; 99 % CI *−*6·0, −1·6) compared with those who did not. Relative importance of reasons for food outlet selection differed by large (price, food quality) *v*. small (proximity, convenient hours) shopping trip and by outlet type.

**Conclusions:**

Findings contribute to our understanding of food store selection and have implications for potentially relevant retail food intervention settings.

Poor diet quality is common in Canada and the USA^(^ ^1^ ^,^ ^2^ ^)^ and is a primary risk factor for many chronic diseases^(^ ^3^ ^,^ ^4^ ^)^. Diet quality indices typically include common food sources and specific nutrients significantly associated with disease^(^ ^3^ ^)^. More frequent fruit and vegetable (FV) consumption, for instance, reduces risk for certain cancers, CVD, obesity and all-cause mortality^(^ ^5^ ^–^ ^9^ ^)^. In Canada, as in other countries^(^ ^10^ ^)^, residents generally fail to meet recommended daily guidelines for FV^(^ ^11^ ^–^ ^15^ ^)^. In terms of specific nutrients, excess dietary sodium is associated with hypertension, especially in well-designed studies^(^ ^16^ ^)^, and SFA is another nutrient of concern, with national dietary guidelines in both Canada^(^ ^17^ ^)^ and the USA^(^ ^18^ ^)^ encouraging citizens to limit saturated fat intake because of its association with CVD.

Individuals’ dietary behaviours and their downstream health effects are embedded within their social, economic and physical environments^(^ ^19^ ^–^ ^23^ ^)^. The retail food environment is of particular concern because the vast majority of dietary energy consumed in Canada^(^ ^24^ ^–^ ^26^ ^)^ and the USA^(^ ^27^ ^)^ is purchased in food stores. Two major knowledge gaps related to food store patronage and dietary outcomes exist. Currently, very little literature examines how patronage of different types of food store is associated with dietary and weight outcomes. Second, little existing research examines consumer motivation for food store selection. Instead, much of the food environment research to date assumes food store selection is predominantly due to proximity^(^ ^28^ ^)^. Both of these gaps are discussed further below.

Only three studies to date have examined how patronizing different types of food store is associated with diet-related outcomes^(^ ^29^ ^–^ ^31^ ^)^. One study using a non-probability adult sample found that shopping primarily in discount stores and hypermarkets was associated with larger waist circumference (WC), while shopping primarily in organic stores was associated with lower BMI and WC^(^ ^29^ ^)^. Another smaller study of African-American women found that those shopping primarily at supermarkets and specialty stores consumed more FV on average than those who shopped primarily at independent grocers^(^ ^31^ ^)^. Finally, a study of low-income African-American adults found that frequent corner-store shoppers procured unhealthy foods more frequently than frequent supermarket shoppers^(^ ^30^ ^)^. Of these three studies only one study used a probability sample^(^ ^31^ ^)^ and that study focused exclusively on African-American women in Detroit. None of these few existing investigations^(^ ^29^ ^–^ ^31^ ^)^ reported on associations between shopping at a broad range of food stores and dietary outcomes, and only one study^(^ ^31^ ^)^ used a probability sample.

Second, food environment research often implicitly assumes proximity is the predominant consumer motivator for food store selection^(^ ^28^ ^)^ and few studies have examined consumer motivations for food store choice. Relevant research has found reasons for food store selection typically include considerations of proximity, price and food quality^(^ ^28^ ^,^ ^30^ ^,^ ^32^ ^–^ ^36^ ^)^, as well as availability of specific foods^(^ ^28^ ^,^ ^30^ ^,^ ^32^ ^–^ ^34^ ^,^ ^36^ ^)^, store neighbourhood safety^(^ ^28^ ^,^ ^32^ ^–^ ^35^ ^)^ and store cleanliness^(^ ^28^ ^,^ ^30^ ^,^ ^33^ ^)^. All existing studies have been conducted in the USA and the majority of studies have been undertaken with low-income participants^(^ ^30^ ^,^ ^32^ ^,^ ^34^ ^–^ ^36^ ^)^. Only one used randomized participant selection in its study design^(^ ^37^ ^)^. Understanding consumer motivations for food store choice is important for public health researchers and practitioners to effectively design, implement and evaluate real-world retail food interventions.

The objectives of the current study were to: (i) examine associations between the frequency of shopping at different store types and diet and weight-related outcomes; and (ii) explore motivations for food store choice. We fill existing gaps in the literature by using a large, representative sample of residents from three mid-sized urban municipalities in south-western Ontario, by examining a range of dietary and weight-related outcomes, and by examining many different types of food store rather than only supermarkets. While individual attributes and preferences no doubt influence selection of shopping destinations, in-store marketing, food availability, affordability and quality also influence consumer purchasing decisions that have implications for long-term dietary health^(^ ^38^ ^,^ ^39^ ^)^.

## Methods

Between May 2009 and May 2010, the NEWPATH (Neighbourhood Environments in Waterloo Region: Patterns of Transportation and Health) study assessed food shopping behaviours, dietary intake and built environment features in three spatially contiguous cities (Kitchener, Cambridge and Waterloo) within the Region of Waterloo, Ontario^(^ ^40^ ^)^. Proportional sampling was used to recruit a stratified, random, representative sample of tri-city residents. Telephone numbers of households within eligible postal codes (based on walkability) were purchased from the firm ASDE Survey Sampler and a telephone recruitment survey was used to randomly select potential participants according to the following sample stratification criteria: neighbourhood walkability (high, medium and low), household income and household size. Participants were recruited to be representative of the study area in terms of income and household size within walkability strata according to 2006 census data. The conditional response rate (response rate once a household agreed to participate) was 61 %. All household residents over 10 years of age in participating households completed either a ‘simple’ or an ‘enhanced’ survey package. The simple package included a household paper survey, which was completed by the self-identified head of household, and an individual-level two-day travel diary, which was completed by all participants (all household residents over 10 years of age). All participants reported their height, weight and WC according to a standard protocol^(^ ^41^ ^)^. The main food shopper in each household completed an additional food shopping survey. Randomly selected enhanced survey participants were a subset of participants who completed the simple survey. These participants were additionally asked to complete a prospective two-day food diary as part of the two-day travel diary. Enhanced travel diaries included questions about purchasing and eating or drinking at each place. For each travel destination, participants provided a detailed description of the foods and/or drinks consumed, the amount consumed and other details about the items (e.g. percentage fat in milk, product brand). Common measures and guides to estimate portion sizes (e.g. 1 cup (250 ml)=size of a tennis ball) were included as a folding flap in each enhanced travel diary. In total, data from 4574 individuals within 2596 households were obtained. Of these, a subset of 1362 individuals within 755 households participated in the enhanced survey. The sub-sample of participants who completed the enhanced survey package had similar sociodemographic characteristics and outcomes to the full sample.

The NEWPATH study received ethics clearance from the University of Waterloo Office of Research Ethics and the University of British Columbia’s Behavioural Research Ethics Board.

### Measures

#### Frequency of patronizing different types of food store

The main food shopper in each household was asked, ‘When you go shopping for household food purchases, how often do you and/or other household members go to the following types of places?’ for the following places: supermarket; supercentre store; convenience store; specialty store; farmers’ market; food bank; home delivery; and food co-op. To increase participant understanding and consistency in reporting, for each store type, local examples of stores of that type were included in the survey. Response options (‘never/rarely’, ‘less than one time per month’, ‘about one time per month’, ‘about two times per month’, ‘about one time per week’, ‘two or more times per week’) were categorized into frequent (once per week or more) and infrequent (two times per month or less). For home delivery, food banks and food co-ops, response options were categorized as frequent (once per month or more) and infrequent (less than once per month) because of the low prevalence of respondents who reported getting food from home delivery (0·5 %), a food bank (0·3 %) or from food co-ops (0·5 %) at least once per week. Missing data ranged from sixty-six missing responses for supermarket shopping frequency (2·5 %) to 144 missing responses for food bank use frequency (5·5 %).

#### Reasons for patronizing food stores

The main food shopper in each household was asked, ‘For each type of place you shop at, please rank the top three reasons why you choose a specific type of store when you plan to buy a LARGE amount of food’. The full list of reasons as administered in the survey included: cheaper prices/accept coupons/specific sale day; close to home, work, school or daily activities; convenience services (e.g. grocery packing, pickup, parking, seated grocery carts); quality of fresh produce, meat or bread; convenient hours of operation; they have foods that other stores do not carry; I like to buy local; I know the vendor; buying in bulk; and for personal, ethical or religious reasons. This question was repeated for small grocery shopping trips. Potential reasons for food store choice were selected based on existing literature in 2008 when the survey items were developed and stakeholder consultations, where local stakeholders and consumers were invited to provide potential reasons for food store choice.

#### Frequency of consumption of fruit and vegetables

The six-item FV consumption frequency measure was adapted from the Canadian Community Health Survey^(^ ^42^ ^,^ ^43^ ^)^. FV consumption frequency was included as an outcome of interest to compare these local results with national averages. Participants were asked how many servings they usually ate of 100 % juice, whole fruit, green salad, carrots, potatoes (not fried) and other vegetables. The frequency of FV consumption was calculated as the number of times FV were consumed per day^(^ ^42^ ^,^ ^43^ ^)^. While FV screeners are typically unable to provide precise estimates of FV intake^(^ ^44^ ^)^, this screener was included in the current study to compare FV intake in the Region of Waterloo with national estimates for key stakeholders.

#### BMI and waist circumference

BMI was calculated based on self-reported weight and height (kg/m^2^). Mean WC was obtained from two self-assessed measurements (to the nearest centimetre). Each survey package included a paper measuring tape, which participants used to record their WC twice, according to a standard protocol^(^ ^41^ ^)^. Self-reported WC obtained via this protocol has been shown to be significantly over-reported by approximately 1cm, but there is high concordance between self-reported and measured values (intraclass correlation coefficient=0·96; 95 % CI 0·94, 0·97)^(^ ^41^ ^)^.

#### Sociodemographic variables

Participants reported sociodemographic variables including age, sex, household income (which was categorized as low (<$CAN 35000/year), medium ($CAN 35000–85 000/year) and high (>$CAN 85000/year)), household size (the number of people in the household) and education (which was classified as low (high school or below), medium (some university or college) and high (completed post-secondary education)).

#### Diet quality and dietary components

Data from two 24 h food records on subsequent days were entered into nutrition analysis software (ESHA Food Processor SQL™ version 8·5), which provided overall energy, macro- and micronutrient estimates for each day. Diet quality was assessed using the Healthy Eating Index score adapted for Canada (HEI-C), which assesses dietary adequacy and moderation based on age- and sex-specific recommendations from Canada’s Food Guide^(^ ^2^ ^)^. The HEI-C was computed based on standard protocol^(^ ^2^ ^)^ and could range from 0 to 100, with higher scores representing better diet quality. Mean energy (kcal), sodium (mg), saturated fat (g) and HEI-C scores over the two days were treated as continuous variables. To maximize validity of dietary data, the National Cancer Institute recommends dietary data derived from multiple administrations of a 24 h recall (or food record) for studies examining associations between independent variables and dietary outcomes as a dependent variable^(^ ^45^ ^)^.

### Statistical analyses

Descriptive statistics were used to examine the frequency of patronizing different types of food store and the reasons for patronizing different outlet types for large and small shopping trips. Data from respondents to the simple and enhanced surveys were analysed together, except for dietary data derived from travel diaries, which were analysed only for the enhanced survey respondents. Generalized estimating equation models, which account for the nested structure of the data (individuals nested within households), were used to examine associations between frequency of patronizing different types of food store and dietary and weight-related outcomes. Age, sex, household income, household size and education were included as covariates in all models.

The statistical software package SAS^®^ version 9·3 was used for all analyses; PROC GENMOD was used to create the generalized estimating equation models. PROC SURVEYREG using households as primary sampling units was also run for analyses with continuous outcome variables for comparison. Both sets of analyses provided almost identical estimates and *P* values. Estimates from the generalized estimating equation models are presented here. BMI and WC variables were slightly skewed and a two-step transformation was used to improve variable normality^(^ ^46^ ^)^. Dietary analyses excluded outliers (those with implausible energy intake, defined as those who reported consuming <418·4 kJ (<100 kcal) or >41840 kJ (>10000 kcal) per day, *n* 21). Similarly, those with BMI>50 kg/m^2^ were excluded from analyses as outliers (*n* 16). Data from participants with complete data on all variables of interest were included in each analysis (exact analytic sample sizes are noted in results tables). Pairwise deletion was used to increase power of the tests by maximizing all data available on an analysis-by-analysis basis. The proportion of complete cases ranged from 85 % (BMI analyses) to 99 % (energy (kcal), saturated fat and sodium intake analyses). Survey weights were constructed for participating households and individuals. Basic inflation survey weights were the reciprocals of the inclusion probabilities. Household weights were then calibrated to sum to assumed totals in quota cells from 2006 census data (the most recent census data available at the time), while the individual weights were calibrated to sum to assumed totals for sex and age group from 2006 census data for the Kitchener Census Metropolitan Area. Data were weighted to reflect census 2006 proportions within walkability areas. A stringent *P*<0·01 was considered statistically significant to account for the possibility of falsely rejecting the null hypothesis given that multiple tests were conducted. Data were analysed in 2015.

## Results

Sample characteristics of the full sample and the sample completing two-day food records (complex sample) are presented in Table 1.

**Table 1:** Sample characteristics, NEWPATH study, Region of Waterloo, Ontario, Canada, 2009/2010

	Full sample (*n* 4574)	Complex sample (*n* 1362)
Sex		
Female	54·2	55·2
Male	45·8	44·9
Annual household income ($CAN)		
High (>85 000)	40·8	47·7
Medium (35 000–85 000)	41·9	36·0
Low (<35 000)	17·3	16·3
Mean age (years)	42·8	42·1
sd	18·1	17·6
Education level		
High school or below	32·5	26·3
Partial college or university	9·9	10·6
Completed post-secondary	57·6	63·1
Mean HEI-C score	n/a	53·3
sd	–	9·8
Mean FV intake frequency (per d)	5·2	5·3
sd	2·8	2·8
Mean energy intake (kJ/d)	n/a	7457
sd	–	3516
Mean energy intake (kcal/d)	n/a	1782·3
sd	–	840·4
Mean Na intake (mg/d)	n/a	3113·7
sd	–	1917·2
Mean saturated fat intake (g/d)	n/a	26·7
sd	–	14·6
Mean BMI (kg/m^2^)	27·6	27·0
sd	5·9	6·0
Mean WC (cm)	89·8	89·4
sd	16·0	16·2

HEI-C, Healthy Eating Index for Canada; FV, fruit and vegetable; WC, waist circumference; n/a, not applicable. Data are presented as weighted mean and standard deviation or percentage.

### Frequency of patronizing different types of food store

The percentage of participants who shopped at least once per week at each type of food store was 90·6 % for supermarkets, 16·1 % for supercentres, 10·4 % for convenience stores, 7·9 % for specialty stores, 7·4 % for farmers’ markets, 0·5 % for home delivery, 0·5 % for food co-ops and 0·3 % for food banks (see Table 2).

**Table 2:** Weighted* percentage of frequency of shopping at different types of food store (*n* 2596), NEWPATH study, Region of Waterloo, Ontario, Canada, 2009/2010

	No. of participants who report shopping at each food store†	%	Never/rarely	Less than once per month	Once per month	Twice per month	Once per week	At least twice per week
Supermarket	2526	99·8	0·1	0·5	1·0	7·7	47·8	42·8
Supercentre	1923	77·8	18·1	24·0	23·8	17·9	13·3	2·8
Convenience store	1123	45·9	54·0	18·0	9·6	8·0	6·3	4·1
Specialty store	1569	63·7	40·0	25·8	15·7	10·6	6·3	1·6
Farmers’ market	1700	68·6	34·5	31·7	14·8	11·7	6·7	0·7
Food bank	47	1·9	97·4	1·7	0·1	0·4	0·3	0·0
Home delivery	122	5·0	95·3	2·4	0·8	1·0	0·5	0·0
Food co-op or informal buying group	72	2·9	98·0	1·1	0·3	0·1	0·5	0·0

* Percentages were weighted by the household inflation weights to account for sample stratification and to represent the population of the three cities according to walkability of the neighbourhood, household size and household income.

† Percentages are among respondents who had complete data for survey items (i.e. they do not include missing values). Missing values ranged from sixty-six missing responses for supermarkets (2·5 %) to 144 missing responses (5·5 %) for food banks.

### Reasons for patronizing different types of food store for large and small shopping trips

Table 3 provides the percentage of main food shoppers choosing each reason as one of their top three reasons for food store selection by food store type for large and small shopping trips. For large food shopping trips, the most popular reasons for choosing a specific supermarket were proximity, food quality and price. For supercentres, price was the most commonly reported reason, followed by convenient hours and proximity. Respondents commonly reported choosing convenience stores based on proximity (85·8 %) and convenient hours (83·8 %). Participants reported choosing farmers’ markets because of food quality (90·5 %) and to ‘buy local’ (77·8 %). Sale of specific foods and food quality were the most commonly reported reasons for selecting specialty stores.

**Table 3:** Weighted percentage of main food shoppers choosing each reason as a ‘top three’ reason for food store selection for large and small shopping trips among those who responded that they do patronize that type of store (*n* 2596), NEWPATH study, Region of Waterloo, Ontario, Canada, 2009/2010

	Price	Proximity	Convenience services	Quality	Convenient hours	Specific foods	Buy local	Know vendor	Buy bulk	Personal or ethical
Large shopping trip										
Supermarket	60·6	74·5	24·8	67·3	42·1	17·9	7·2	1·4	3·3	1·2
Supercentre	88·8	39·7	22·1	13·0	45·7	28·0	2·1	1·5	36·4	2·3
Convenience store	12·6	85·8	32·2	3·4	83·8	7·4	10·6	11·0	2·3	5·2
Specialty store	13·9	25·8	6·4	64·2	5·3	79·9	27·6	10·8	7·3	17·8
Farmers’ market	25·0	17·0	1·8	90·5	3·3	36·6	77·8	10·8	9·2	5·2
Food bank	59·8	31·2	28·4	19·6	41·8	20·0	19·3	20·3	11·0	57·8
Home delivery	21·8	20·9	48·3	29·5	57·3	19·8	15·6	8·0	16·8	28·7
Food co-op	60·2	17·3	0·0	40·7	21·2	50·7	49·1	26·8	24·1	10·4
Small shopping trip										
Supermarket	54·0	80·9	31·7	48·9	57·1	14·8	7·7	2·1	2·2	2·0
Supercentre	82·1	51·1	32·2	13·9	54·1	23·9	4·5	1·8	16·8	2·2
Convenience store	18·5	86·2	40·0	5·1	91·7	9·1	7·5	12·5	1·0	3·6
Specialty store	10·9	30·0	7·9	61·4	6·8	80·6	33·8	11·2	2·5	16·8
Farmers’ market	24·0	17·7	3·9	86·5	5·2	43·4	73·9	9·3	6·2	6·3
Food bank	49·5	52·3	52·8	28·2	35·9	27·2	29·3	29·8	26·9	69·1
Home delivery	32·0	36·1	48·5	16·7	52·8	30·7	15·4	3·5	3·0	25·0
Food co-op	28·9	15·8	11·8	56·0	11·8	30·6	56·6	32·0	11·3	44·4

### Associations between frequency of patronizing different types of food store and diet and weight-related outcomes

Table 4 presents parameter estimates and 99 % CI of dietary and weight-related outcomes associated with frequently (at least once per week) patronizing various store types. After adjusting for covariates, participants who frequently shopped at food co-ops (*β*=5·3; 99 % CI 0·3, 10·2) had significantly higher diet quality than people who did not. All other diet quality comparisons were non-significant at *P*<0·01.

**Table 4:** Parameter estimates and 99 % CI of dietary and weight-related outcomes associated with frequently (at least once per week) patronizing various store types, NEWPATH study, Region of Waterloo, Ontario, Canada, 2009/2010

	Adjusted estimates*																				
	Diet quality (HEI-C) (*n* 1330)			FV frequency† (*n* 2596)			Mean energy (kcal) (*n* 1346)			Mean sodium (mg) (*n* 1346)			Mean saturated fat (g) (*n* 1346)			BMI† (kg/m^2^) (*n* 3887)			WC (cm) (*n* 3939)		
Variable	*β*	99 % CI	*P* value	*β*	99 % CI	*P* value	*β*	99 % CI	*P* value	*β*	99 % CI	*P* value	*β*	99 % CI	*P* value	*β*	99 % CI	*P* value	*β*	99 % CI	*P* value
Supermarket	1·1	−1·5, 3·7	0·2665	0·6	0·03, 1·2	0·0069	86·8	−219·6, 393·2	0·4655	189·3	−466·5, 845·0	0·4573	−14·0	−51·1, 23·0	0·3290	−0·20	−1·29, 0·88	0·6314	−0·16	−3·00, 2·66	0·8776
Convenience store	−3·4	−6·9, 0·2	0·0151	−0·6	−1·1, −0·02	0·0072	139·8	−209·7, 489·2	0·3030	−324·7	−2313·1, 1663·6	0·6740	−1·7	−11·8, 8·4	0·6603	0·72	−0·37, 1·81	0·0877	1·61	−1·21, 4·42	0·1417
Supercentre	−1·9	−4·7, 0·9	0·0788	−0·2	−0·7, 0·2	0·1632	−133·6	−431·3, 164·1	0·2478	−714·8	−2272·9, 843·2	0·2373	−4·1	−13·0, 4·8	0·2368	0·89	−0·02, 1·80	0·0116	1·78	−0·56, 4·12	0·0496
Specialty store	2·3	−0·1, 4·6	0·0142	1·3	0·6, 1·9	<0·0001	−10·8	−261·1, 239·5	0·9116	−422·5	−1885·7, 1040·8	0·4570	−3·3	−9·8, 3·2	0·1987	−2·08	−3·04, −1·13	<0·0001	−4·79	−7·04, −2·54	<0·0001
Farmers’ market	1·3	−1·3, 3·9	0·2041	0·9	0·3, 1·6	0·0003	76·8	−175·5, 329·2	0·4328	3724·4	−5446·1, 12894·8	0·2955	6·8	−12·4, 26·0	0·3633	−1·39	−2·28, −0·51	<0·0001	−3·83	−6·02, −1·63	<0·0001
Home delivery‡	−0·9	−7·3, 5·5	0·7159	−1·3	−2·3, −0·4	0·0003	68·9	−468·7, 606·6	0·7412	623·8	−1303·4, 2551·0	0·4044	7·5	−9·7, 24·8	0·2607	2·21	−0·21, 4·64	0·0185	5·90	−0·90, 12·70	0·0254
Food bank‡	−1·5	−25·4, 22·3	0·8686	−1·6	−2·9, −0·3	0·0017	−418·3	−1734·0, 897·5	0·4129	−764·9	−4105·4, 2575·6	0·5553	−14·4	−27·8, −1·0	0·0057	−3·05	−8·96, 2·86	0·1835	−4·30	−21·47, 12·87	0·5190
Food co-op or CSA‡	5·3	0·3, 10·2	0·0062	1·6	0·1, 3·1	0·0055	230·1	−105·0, 565·1	0·0769	−686·9	−2232·4, 858·7	0·2523	−0·6	−6·0, 4·9	0·7917	−0·76	−2·82, 1·31	0·3457	−4·16	−8·05, −0·27	0·0059

HEI-C, Healthy Eating Index for Canada; FV, fruit and vegetable; WC, waist circumference; CSA, community-supported agriculture. All generalized linear models accounted for sex, whether or not participants completed education beyond high school, age, household income and household size. Models examining diet quality, mean energy (kcal), mean sodium and mean saturated fat were based on the sub-sample of participants who completed two-day food records and were weighted by individual-level weights to ensure generalizability of the sample. Analyses examining FV consumption used a household-level weight, since FV consumption was reported at the household level.

† FV frequency was a household-level outcome, therefore models were at the household level, so analyses did not cluster based on household.

‡ Because of the small number of people who reported accessing food from the food bank or food co-op at least once per week, the frequency of food purchasing from these two outlets was defined as 0 = less than once per month; 1 = once per month or more.

Participants who shopped frequently at supermarkets (*β*=0·6; 99 % CI 0·03, 1·2), specialty stores (*β*=1·3; 99 % CI 0·6, 1·9), farmers’ markets (*β*=0·9; 99 % CI 0·3, 1·6) and food co-ops (*β*=1·6; 99 % CI 0·1, 3·1) consumed FV significantly more frequently than people who did not. Those who shopped frequently at convenience stores (*β*=−0·6; 99 % CI −1·1, −0·02), used home delivery services (*β*=−1·3; 99 % CI −2·3, −0·4) and used food banks frequently (*β*=−1·6; 99 % CI −2·9, −0·3) consumed FV less frequently than those who did not.

Frequent specialty store and farmers’ market shoppers had significantly lower BMI (*β*=−2·08; 99 % CI −3·04, −1·13 and *β*=−1·39; 99 % CI −2·28, −0·51, respectively) and WC (*β*=−4·79; 99 % CI −7·04, −2·54 and *β*=−3·83; 99 % CI −6·02, −1·63, respectively) than those who did not shop at these stores frequently. Frequent food co-op shoppers had significantly lower WC (*β*=−4·16; 99 % CI −8·05, −0·27) compared with those who did not.

## Discussion

Frequency of patronizing different food outlets was significantly associated with dietary and weight-related outcomes in this representative sample of residents from three mid-sized urban municipalities in Ontario. The current study makes three major contributions to the literature. First, it showed significant associations between dietary and weight-related outcomes and the frequency of patronizing different types of food store. Second, it examined a wider variety of food store types than has been examined in previous studies. Finally, it is among the first studies to examine the reasons people report patronizing different types of food outlet for both large and small grocery shopping trips.

### Associations between food store patronage and dietary and weight-related outcomes

First, people who shopped frequently at convenience stores consumed FV less frequently compared with people who did not shop at these outlets frequently, even after controlling for age, sex, education and income. These findings support previous research that frequent food shopping at corner stores is associated with unhealthy food procurement practices^(^ ^30^ ^)^. Corner stores tend to be settings in which energy-dense, non-nutritious foods and beverages are readily available^(^ ^39^ ^,^ ^47^ ^,^ ^48^ ^)^. Although only 10 % of participants patronized corner stores at least once per week in our sample, corner stores may nevertheless represent important settings in which to intervene to improve public health^(^ ^48^ ^)^. In contrast, frequent supermarket and specialty store shoppers consumed FV more frequently than those who did not, similar to findings from previous research^(^ ^31^ ^)^. Frequent supercentre shoppers had marginally higher mean BMI than those who did not shop there frequently, consistent with findings that people shopping in hypermarkets had significantly higher BMI and WC relative to those who shopped in city markets^(^ ^29^ ^)^. The latter study found that participants shopping in organic shops had significantly lower BMI and WC compared with those who shopped in regular supermarkets^(^ ^29^ ^)^, consistent with our finding that frequent specialty store shoppers had significantly lower BMI and WC compared with those who infrequently shopped at specialty stores.

### Food outlet patronage in south-western Ontario

Second, the present study found that although 91 % of respondents reported shopping at least once per week in a supermarket, other retail food sources may also contribute substantially to dietary intake at a population level. A sizeable percentage of respondents reported shopping at least twice per month in supercentres (34 %), farmers’ markets (19 %), specialty stores (19 %) and convenience stores (18 %). Given the significant associations between frequency of shopping at each of these food sources and diet- and/or weight-related outcomes found in the study, future research should examine consumer behaviour in these types of food store to develop appropriate interventions for various retail food contexts. For example, future work could build on current supermarket initiatives like the Guiding Stars campaign^(^ ^49^ ^)^ to create context-specific interventions for a broader variety of food store types.

### Consumer motivations for food store selection

Third, these findings contribute to the small but growing public health literature on reasons for food store selection. The most popular reasons for food outlet choice for large grocery shopping trips included price, food quality and proximity. For smaller grocery shopping trips, proximity and convenient hours were the most frequently cited reasons people chose a particular shopping destination. Store proximity is only one of many factors involved in food store selection^(^ ^28^ ^,^ ^32^ ^)^. Of note, we found that proximity was not universally important to consumers in selecting a specific food source. For example, 86 % of convenience store shoppers cited the importance of proximity in store selection compared with 75 % of supermarket shoppers, 26 % of specialty store shoppers and only 17 % of farmers’ market shoppers. Additionally, proximity was more frequently cited as an important reason for food store selection for small grocery shopping trips compared with large grocery shopping trips. This indicates that for consumers, proximity varies in relevance based on store type and shopping trip context. This finding has important implications also for the field of food environments research, given that access to food retailers is often operationalized as geographic proximity^(^ ^50^ ^,^ ^51^ ^)^. Understanding why people choose the food outlets they do, and how these factors may vary by food outlet type, is important for advancing food environment research and interventions.

### Strengths and limitations

Strengths of the present study include the representative sample and relatively high response rates, assessment of a broad variety of food store types and motivations for visiting each one, and inclusion of two weight-related measures (BMI and WC), especially since WC is superior to BMI in predicting mortality risk^(^ ^52^ ^)^. Limitations of the study include the potential for selection bias. For example, it is possible that individuals with healthier diets choose to shop more frequently at specialty stores. Certainly, the cross-sectional nature of the current study means we cannot assume directionality of findings. However, given that in-store marketing also influences consumers’ food purchasing^(^ ^38^ ^,^ ^47^ ^,^ ^53^ ^,^ ^54^ ^)^, it is likely that the link between dietary outcomes and food store patronage is actually bidirectional. Another limitation was the use of self-reported dietary and weight-related outcomes. Although respondents generally overestimate height and underestimate weight^(^ ^55^ ^,^ ^56^ ^)^, health risks associated with variations in self-reported BMI are comparable to those associated with variations in measured BMI^(^ ^57^ ^)^. Also, average WC for men and women were in line with national objective estimates from the same time frame^(^ ^58^ ^)^, although our study’s BMI estimates were higher than national self-report estimates for both males (28·3 kg/m^2^ in our study, 26·7 kg/m^2^ nationally) and females (27·4 kg/m^2^ in our study, 25·3 kg/m^2^ nationally)^(^ ^59^ ^)^. Our questions did not specify what type of supermarket residents patronized (i.e. discount *v*. regular). Patrons of discount supermarkets may be more likely to have higher BMI than patrons of regular supermarkets^(^ ^29^ ^,^ ^60^ ^)^. This limitation may help to explain the null associations between frequent supermarket shopping and weight outcomes. Alternatively, the high prevalence of frequent supermarket shoppers may have resulted in this variable not being sufficiently discriminatory in the current sample. It is also possible that overall shopping frequency (e.g. many small food shopping trips throughout the week *v*. one large shopping trip less frequently) may contribute to dietary and weight outcomes. Unfortunately, the structure of our shopping frequency survey item rendered it impossible to derive an overall shopping frequency variable. Finally, we attempted to define shopping frequency variables consistently where possible (e.g. at least once per week *v*. less than once per week) instead of dichotomizing at the median for each frequency variable. Dichotomizing at the median would have resulted in a more even sample between frequent and infrequent shoppers, but may not have been as meaningful. For example, 54 % of respondents reported never or rarely shopping at convenience stores. Had we defined infrequent convenience store shopping as ‘never or rarely,’ frequent convenience store shopping would have been defined as ‘less than once per month or more’, which is likely not a theoretically meaningful definition of frequent convenience store shopping. Unfortunately, the low numbers of participants who patronized food banks, home delivery shopping and food co-ops precluded the possibility of defining frequent shopping as at least once per week for all outlets. Therefore, future research should examine how more frequent use of food banks, home delivery services and food co-ops is associated with dietary and weight-related outcomes. Future research should also examine overall shopping frequency as it pertains to dietary and health outcomes.

## Conclusions

Findings from the current study may be generalizable to other mid-sized North American cities. Importantly, findings indicate that corner stores and supercentres may both be important public health nutrition intervention settings, given frequent shopping at these locations is associated with poorer dietary and weight-related outcomes. In addition, these findings can help to further refine measures of access in food environments research, in particular by taking into account features of the consumer nutrition environment related to food prices and food quality.
